# Designing Waveform Sets with Good Correlation and Stopband Properties for MIMO Radar via the Gradient-Based Method

**DOI:** 10.3390/s17050999

**Published:** 2017-05-01

**Authors:** Liang Tang, Yongfeng Zhu, Qiang Fu

**Affiliations:** College of Electronic Science and Engineering, National University of Defense Technology, Changsha 410073, China; zoyofo@163.com (Y.Z.); fuqiang1962@vip.sina.com (Q.F.)

**Keywords:** multiple-input multiple-output (MIMO) radar, unimodular waveform set, auto- and cross-correlation, frequency stopband, gradient

## Abstract

Waveform sets with good correlation and/or stopband properties have received extensive attention and been widely used in multiple-input multiple-output (MIMO) radar. In this paper, we aim at designing unimodular waveform sets with good correlation and stopband properties. To formulate the problem, we construct two criteria to measure the correlation and stopband properties and then establish an unconstrained problem in the frequency domain. After deducing the phase gradient and the step size, an efficient gradient-based algorithm with monotonicity is proposed to minimize the objective function directly. For the design problem without considering the correlation weights, we develop a simplified algorithm, which only requires a few fast Fourier transform (FFT) operations and is more efficient. Because both of the algorithms can be implemented via the FFT operations and the Hadamard product, they are computationally efficient and can be used to design waveform sets with a large waveform number and waveform length. Numerical experiments show that the proposed algorithms can provide better performance than the state-of-the-art algorithms in terms of the computational complexity.

## 1. Introduction

Waveform design has received considerable attention in recent years [1] and been employed in many applications, including polarimetric radar [2], multiple-input multiple-output (MIMO) radar [3,4], stealth communications [5] and the code-division multiple-access (CDMA) system. As a category of the general waveform design research [1], the design of waveform sets (i.e., multidimensional waveforms) is an important research content of MIMO radar. Generally, waveform sets are desired to have a good correlation property, which can effectively improve radar resolution, detection performance, imaging quality, the ability to obtain information and the accuracy of MIMO channel estimation [6,7,8]. In recent years, a large number of scholars has been devoted to designing waveform sets with a good correlation property. The main research covers two aspects: one is the waveform sets with good auto- and cross-correlation properties [9,10,11,12,13,14,15,16], and the other is the complementary sets of sequences (CSS) [17,18,19,20,21,22,23,24,25].

Waveform sets with good auto- and cross-correlation properties, also known as the orthogonal waveform set (OWS), have low autocorrelation sidelobes and low cross-correlation levels. In the early stage, the simulated annealing- [9] and cross entropy-based [10] methods were proposed for OWS design. However, due to the high computational complexity, these methods are not suited to design long waveforms. To improve the computational efficiency, the CAN (cyclic algorithm-new) algorithm [11] based on fast Fourier transform (FFT) is proposed to minimize the autocorrelation sidelobes and the cross-correlation. This algorithm is computationally efficient and can be used for the design of long waveforms. As it is impossible to design completely orthogonal (i.e., both the autocorrelation sidelobes and the cross-correlation are zeroes) waveform sets [11,16], [11] proposes to design the waveform sets that are orthogonal only at the specified intervals and extends the classical WeCAN (weighted cyclic algorithm-new) [26] algorithm to MIMO radar. To solve the same problem, [12] develops the LBFGS (limited-memory Broyden, Fletcher, Goldfarb and Shanno) iterative algorithm, which is more efficient than the WeCAN algorithm. However, because of the complicated linear search rule for determining the step size, the LBFGS iterative algorithm is still time consuming. Recently, the majorization-minimization (MM)-based algorithms (i.e., MM-Corr (MM-correlation) and MM-WeCorr (MM-weighted correlation)) are proposed in [14]. These two algorithms are also based on FFT operations and much faster than the CAN and WeCAN algorithms [11].

Another waveform set with a good correlation property is the complementary sets of sequences (CSS). A waveform set is called CSS if and only if the autocorrelation sum of the waveforms is a delta function [17]. CSS design is proposed to overcome the difficulty of generating a single unimodular waveform with ideal (impulse-like) autocorrelation. A common application of the CSS is the pulse compression [18,19,20]. In pulse compression radar, the complementary sequences are used to modulate consecutive pulses in a coherent pulse train. Then, the autocorrelation sidelobes can be reduced via the coherent [19] or noncoherent [18] accumulation, which can be regarded as the process of obtaining the autocorrelation sum of the complementary sequences. Moreover, due to the good correlation property, CSS has been widely applied to the CDMA system [21], ISI (intersymbol interference) channel estimation [22], orthogonal frequency division multiplexing (OFDM) [23], and many other areas. The main methods of designing CSS are the analytical construction methods, which have great limitation in generating long waveforms. To overcome this problem, [24] introduces a computational framework based on an iterative twisted approximation (ITROX) for periodically complementary sets of sequences design. Subsequently, [25] extends the CAN algorithm [26] and develops a fast algorithm named CANARY (CAN complementary). Additionally, [14] applies the MM method to the design of CSS.

In addition to the good correlation property, waveform sets are expected to have a good stopband property when the radar systems work in a crowded electromagnetic environment. Waveforms with the stopband property, also known as the sparse frequency waveforms (SFW) in many literature works, are a kind of waveforms with several frequency stopbands. The applications of SFW include ultra-wide bandwidth (UWB) systems [27], high frequency surface wave radar (HFSWR) [28,29] and cognitive radar [30]. By designing waveforms with the stopband property, it can effectively overcome the narrowband interference in the congested frequency bands. At present, there are many research works on the design of a single waveform with the stopband property [31,32,33,34,35,36], but they cannot be used in MIMO systems. Therefore, [36] proposes an iterative algorithm combined with the steepest descent (SD) method for MIMO waveform design. By searching along the gradient direction, the convergence speed of this algorithm is improved. However, the computation of the step size along the gradient direction is complicated, which makes the algorithm still costly. In order to improve the computational efficiency, [37] proposes an algorithm named MDISAA-SFW (multi-dimensional iterative spectral approximation algorithm-SFW) based on alternating projection and phase retrieval.

In this paper, we consider the problem of designing unimodular waveform sets with good correlation and stopband properties and propose a gradient-based algorithm, i.e., Gra-WeCorr-SFW (gradient-weighted correlation-SFW). By using the relationship between the correlation function and the power spectrum density (PSD), the design problem is formulated as an unconstrained minimization problem in the frequency domain. Then, the phase gradient is deduced, and its matrix form is given. In order to avoid searching the step size, we use the Taylor series expansion to derive the step size, which is more efficient than the traditional searching methods. Since both the gradient and the step size can be implemented via the FFT operations and the Hadamard product, the proposed algorithm has high computational efficiency. We also deduce the simplified algorithm named Gra-Corr-SFW (gradient-correlation-SFW), which is faster than the Gra-WeCorr-SFW, for the design problem without considering the correlation weights.

The rest of the paper is organized as follows. In Section 2, the design problem is formulated. In Section 3, we develop a gradient-based algorithm by deducing the phase gradient and the step size and then summarize the algorithm. In Section 4, the simplified algorithm for the design problem without considering the correlation weights is derived. Section 5 provides several numerical experiments to verify the effectiveness of the proposed algorithms. Finally, Section 6 gives the conclusions.

Notation: Boldface upper case letters denote matrices, while boldface lower case letters denote column vectors. ${\left(\cdot \right)}^{*}$, ${\left(\cdot \right)}^{T}$ and ${\left(\cdot \right)}^{H}$ denote the complex conjugate, transpose and conjugate transpose, respectively. $∥\cdot ∥$ and ${∥\cdot ∥}_{F}$ denote the Euclidean norm and the Frobenius norm. Re(·) and Im(·) denote the real and imaginary part, respectively. Diag(x) denotes a diagonal matrix formed with the column vector x. ∘ denotes the Hadamard product. x(m) denotes the *m*-th element of the vector x. $x^{l}$ is the *l*-th iteration of x. $1_{N}$ is the all-one vectors of length *N*. $I_{N}$ denotes the N×N identity matrix. F(x) and $F^{-1}\left(x\right)$ denote the 2N-point FFT and IFFT (inverse FFT) operations of x, respectively. $F_{c}\left(X\right)$ and $F_{c}^{-1}\left(X\right)$ represent the FFT and IFFT of each column of the matrix X, respectively. In the (I)FFT operations, if the length of x is less than 2N, x is padded with trailing zeros to length 2N. $e^{\left(\cdot \right)}$ is the exponent arithmetic applied to the scalar, vector or matrix.

## 2. Problem Formulation

As mentioned in the Introduction, this paper focuses on the problem of designing waveform sets with good correlation and stopband properties. Therefore, we first establish two criteria in the frequency domain to measure the correlation and stopband properties and then formulate the waveform set design problem.

### 2.1. The Criterion for Good Correlation Property

Let ${\left\{x_{m}\right\}}_{m=1}^{M}$ be the complex waveform set to be designed. The vector form of each waveform can be expressed as:

(1) $$x_{m}={\left[x_{m}\left(1\right),x_{m}\left(2\right),...,x_{m}\left(N\right)\right]}^{T},\;m=1,...,M,$$

where *N* is the waveform length and *M* denotes the number of the waveforms. Then, the correlation function of $x_{i}$ and $x_{j}$ is defined as:

(2) $$\begin{array}{c} r_{ij}\left(k\right)=\sum_{n=k+1}^{N}x_{i}\left(n\right)x_{j}^{*}\left(n-k\right)=r_{ji}^{*}\left(-k\right),\;\;i,j=1,...,M,\;\;k=1-N,...,N-1. \end{array}$$

Here, we consider the design problems of complementary sets of sequences and waveform sets with both good auto- and cross-correlation properties. For the complementary set of sequences, the common criterion is the complementary integrated sidelobe level (CISL) metric [14,25], which is defined as:

(3) CISL=∑k=1−Nk≠0N−1∑m=1Mrmm(k)2.

for the waveform sets with both good auto- and cross-correlation properties, we consider the following more general weighted measure [14]:

(4) $$\psi =\sum_{i=1}^{M}\sum_{j=1}^{M}\sum_{k=1-N}^{N-1}w_{t}\left(k\right){\left|r_{ij}\left(k\right)\right|}^{2}-w_{t}\left(0\right)MN^{2},$$

where $w_{t}\left(k\right)=w_{t}\left(-k\right)\geq 0,k=0,...,N-1$ denote the weights assigned to different time lags. In those specified intervals that are expected to have as low as possible sidelobes, $w_{t}\left(k\right)=1$, else $w_{t}\left(k\right)=0$.

Let:

(5) $$\begin{array}{cc} r_{ij} & ={\left[r_{ij}\left(0\right),r_{ij}\left(1\right),...,r_{ij}\left(N-1\right),0,r_{ij}\left(1-N\right),...,r_{ij}\left(-1\right)\right]}^{T},\\ w_{t} & ={\left[w_{t}\left(0\right),w_{t}\left(1\right),...,w_{t}\left(N-1\right),1,w_{t}\left(1-N\right),...,w_{t}\left(-1\right)\right]}^{T} \end{array}$$

be the correlation vector and the corresponding correlation weight vector, respectively. The Fourier transform of the correlation function $r_{ij}$ (i.e., power spectrum density (PSD)) can be written as:

(6) $$\begin{array}{cc} p_{ij} & =F\left(r_{ij}\right)=Fr_{ij},\\ r_{ij} & =F^{-1}\left(p_{ij}\right)=\frac{1}{2N}F^{H}p_{ij}, \end{array}$$

where F is the 2N×2N discrete Fourier transform (DFT) matrix with the following expression:

(7) $$F\left(m,n\right)=e^{-j\frac{2mn\pi }{2N}},1\leq m,n\leq 2N.$$

According to (5) and (6), (4) can be expressed in the frequency domain as:

(8) $$\psi =\sum_{i=1}^{M}\sum_{j=1}^{M}r_{ij}^{H}D_{t}r_{ij}-w_{t}\left(0\right)MN^{2}=\frac{1}{4N^{2}}\sum_{i=1}^{M}\sum_{j=1}^{M}p_{ij}^{H}FD_{t}F^{H}p_{ij}-w_{t}\left(0\right)MN^{2},$$

where $D_{t}=\mathrm{Diag}\left(w_{t}\right)$. It is worth noting that the value of the N+1-th element of $w_{t}$ has no effect on the objective function. In order to facilitate the derivation below, the N+1-th element of $w_{t}$ is set to be one.

Actually, when $w_{t}\left(k\right)=1,k=1-N,...,N-1$ (i.e., the correlation weights are not taken into account), Criterion (8) is equivalent to Criterion (3) (see Appendix A), which means that Criterion (3) is a special case of Criterion (8). Thus, here, we just consider Criterion (8). By ignoring the constant term in (8), the criterion related to the correlation property is given by:

(9) $$J_{CF}=\frac{1}{4N^{2}}\sum_{i=1}^{M}\sum_{j=1}^{M}p_{ij}^{H}FD_{t}F^{H}p_{ij}.$$

### 2.2. The Criterion for the Good Stopband Property

The waveform set with a good stopband property means that the PSD of each waveform has several frequency stopbands. Without loss of generality, we consider that the frequency is normalized. Define the set of frequency stopbands as:

(10) $$\Omega_{f}=\overset{n_{s}}{\underset{k=1}{\cup }}\left(f_{k1},f_{k2}\right)\subset \left[0,1\right],$$

where $\left(f_{k1},f_{k2}\right)$ denotes one stopband and $n_{s}$ denotes the number of the stopbands. Considering the 2N-point FFT operations, the corresponding point set of the stopband set $\Omega_{f}$ can be expressed as:

(11) $${\bar{\Omega }}_{f}=\overset{n_{s}}{\underset{k=1}{\cup }}\left(2Nf_{k1},2Nf_{k2}\right)\subset \left[0,2N\right].$$

Define the frequency weight vector as:

(12) $$\begin{array}{c} w_{f}={\left[w_{f}\left(1\right),w_{f}\left(2\right),...,w_{f}\left(2N\right)\right]}^{T},\\ w_{f}\left(p\right)=\left\{\begin{array}{cc} 1, & p\in {\bar{\Omega }}_{f}\\ 0, & \mathrm{otherwise} \end{array}\right.. \end{array}$$

As the PSD of each waveform is nonnegative, the criterion related to the stopband property (or PSD) can be formulated as:

(13) $$J_{PSD}=\sum_{i=1}^{M}{∥w_{f}\circ p_{ii}∥}^{2}=\sum_{i=1}^{M}p_{ii}^{H}D_{f}p_{ii},$$

where $D_{f}=\mathrm{Diag}\left(w_{f}\right)$.

### 2.3. The Minimization Problem

In order to obtain good correlation and stopband properties, both $J_{CF}$ and $J_{PSD}$ should be minimized. Thus, the waveform design problem can be regarded as a multi-objective optimization (also known as Pareto optimization) problem, i.e., finding the Pareto optimal solutions that satisfy the constraints. Here, we apply the traditional weighting method to solve the problem and, thus, formulate a single-objective function as follows:

(14) $$J_{T}=\lambda J_{PSD}+\left(1-\lambda \right)J_{CF}=\sum_{i=1}^{M}p_{ii}^{H}Qp_{ii}+\sum_{i=1}^{M}\sum_{j=1}^{M}p_{ij}^{H}Q^{\prime }p_{ij},$$

where λ∈[0,1] is a weight coefficient by which we can balance the relative weight between $J_{CF}$ and $J_{PSD}$, and:

(15) $$Q=\lambda D_{f},\;\;Q^{\prime }=\frac{1}{4N^{2}}\left(1-\lambda \right)FD_{t}F^{H}.$$

When λ=0, (14) is the criterion for designing waveform sets with a good correlation property. Additionally, when λ∈(0,1], (14) becomes the criterion for SFW design. Generally, for maximizing the transmitter efficiency and reducing the requirement to the hardware, the unimodular constraint [38] is required in the waveform design. Therefore, the design problem can be formulated as the following minimization problem:

(16) $$\begin{array}{c} \min_{{\left\{x_{m}\right\}}_{m=1}^{M}}\;\;J_{T}\\ \;\;\;\;\;s.t.\;\;\;\;\left|x_{m}\left(n\right)\right|=1,\;\;n=1,...,N,\;\;m=1,...,M. \end{array}$$

Let $\phi_{m}$ denote the phase vector of $x_{m}$, i.e., $x_{m}={\left[e^{j\phi_{m}\left(1\right)},e^{j\phi_{m}\left(2\right)},...,e^{j\phi_{m}\left(N\right)}\right]}^{T}$. Then, the problem (16) can be reformulated as the following unconstrained problem with regard to ${\left\{\phi_{m}\right\}}_{m=1}^{M}$:

(17) $$\min_{{\left\{\phi_{m}\right\}}_{m=1}^{M}}\;\;J_{T}=\sum_{i=1}^{M}p_{ii}^{H}Qp_{ii}+\sum_{i=1}^{M}\sum_{j=1}^{M}p_{ij}^{H}Q^{\prime }p_{ij}.$$

## 3. Problem Optimization via the Gradient Method

In this section, we optimize Problem (17) by using the gradient-based algorithm, which is able to guarantee that the objective function is monotonically decreasing at each iteration. In the following, we first derive the phase gradient and the step size and then briefly summarize the algorithm.

### 3.1. Phase Gradient

To facilitate the derivation, let $\phi_{m}\left(n\right)=\phi_{mn}$. Before deriving the phase gradient $\nabla_{\phi_{m}}J_{T}$, we first deduce the derivative of $J_{T}$ with respect to the phase $\phi_{mn}$:

(18) ∂JT∂ϕmn=∂∑i=1MpiiHQpii+∑i=1M∑j=1MpijHQ′pij/∂ϕmn=∂pmmHQ+Q′pmm/∂ϕmn+∂∑j=1j≠mMpmjHQ′pmj∑i=1i≠mMpimHQ′pim/∂ϕmn.

Since ∑i=1i≠mMpimHQ′pim=∑i=1i≠mMpmiHQ′*pmi (see Appendix B), (18) can be rewritten as:

(19) ∂JT∂ϕmn=∂pmmHQ+Q′pmm/∂ϕmn+2∑i=1i≠mM∂pmiHReQ′pmi/∂ϕmn.

The first term in (19) can be simplified as:

(20) ∂pmmHQ+Q′pmm∂ϕmn=∂pmmTQ+Q′pmm∂ϕmn=∂pmmT∂ϕmnQ+Q′+Q+Q′Tpmm=2∂pmmT∂ϕmnReQ+Q′pmm,

where the third equality follows from the fact ${\left(Q+Q^{\prime }\right)}^{T}={\left(Q+Q^{\prime }\right)}^{*}$. Let:

(21) ymm=ReQpmmy′mi=ReQ′pmi,i=1,...,M,

then (20) can be expressed as:

(22) $$\begin{array}{cc} \frac{\partial \left(p_{mm}^{H}\left(Q+Q^{\prime }\right)p_{mm}\right)}{\partial \phi_{mn}} & =2\frac{\partial p_{mm}^{T}}{\partial \phi_{mn}}\left(y_{mm}+{y^{\prime }}_{mm}\right)\\ & =2\sum_{k=1}^{2N}\frac{\partial \left(p_{mm}\left(k\right)\right)}{\partial \phi_{mn}}\left(y_{mm}\left(k\right)+{y^{\prime }}_{mm}\left(k\right)\right). \end{array}$$

According to (A4) and (A5), we can deduce the derivative $\partial \left(p_{mm}\left(k\right)\right)/\partial \phi_{mn}$ as follows:

(23) ∂pmm(k)∂ϕmn=∂fm(k)fm*(k)∂ϕmn=2Re∂fm*(k)∂ϕmnfm(k)=2Re−jxm*(n)ej2π2Nnkfm(k).

By substituting (23) into (22), we have:

(24) ∂pmmHQ+Q′pmm∂ϕmn=4Re−jxm*(n)∑k=12Nfm(k)ymm(k)+y′mm(k)ej2π2Nnk.

To compute the second term of (19), we first deduce ∂pmiHReQ′pmi/∂ϕmn(i≠m):

(25) ∂pmiHReQ′pmi∂ϕmn=2Re∂pmiH∂ϕmnReQ′pmi=2Re∑k=12N∂pmi*(k)∂ϕmny′mi(k).

Similarly to the derivation of (23), it is easy to obtain that:

(26) $$\frac{\partial \left(p_{mi}\left(k\right)\right)}{\partial \phi_{mn}}=jx_{m}\left(n\right)e^{-j\frac{2\pi }{2N}nk}f_{i}^{*}\left(k\right),i\neq m.$$

On the basis of (25) and (26), ∂pmiHReQ′pmi/∂ϕmn can be further denoted as

(27) ∂pmiHReQ′pmi∂ϕmn=2Re−jxm*(n)∑k=12Nfi(k)y′mi(k)ej2π2Nnk.

By substituting (24) and (27) into (19), $\partial J_{T}/\partial \phi_{mn}$ can be simplified as:

(28) ∂JT∂ϕmn=4Re−jxm*(n)∑k=12Nfm(k)ymm(k)+∑i=1Mfi(k)y′mi(k)ej2π2Nnk.

Let $z_{m}$ be the inverse FFT (IFFT) of $f_{m}\circ y_{mm}+\sum_{i=1}^{M}f_{i}\circ {y^{\prime }}_{mi}$, i.e.,

(29) $$\begin{array}{cc} z_{m} & =F^{-1}\left(f_{m}\circ y_{mm}+\sum_{i=1}^{M}f_{i}\circ {y^{\prime }}_{mi}\right)\\ z_{m}\left(n\right) & =\frac{1}{2N}\sum_{k=1}^{2N}\;\left(f_{m}\left(k\right)y_{mm}\left(k\right)+\sum_{i=1}^{M}f_{i}\left(k\right){y^{\prime }}_{mi}\left(k\right)\right)e^{j\frac{2\pi }{2N}nk}, \end{array}$$

then $\partial J_{T}/\partial \phi_{mn}$ in (28) becomes:

(30) ∂JT∂ϕmn=8NRe−jxm*(n)zm(n).

By stacking (30) in a vector, the phase gradient $\nabla_{\phi_{m}}J_{T}$ is given by:

(31) ∇ϕmJT=∂JT/∂ϕm1,...,∂JT/∂ϕmNT=8NRe−jxm*∘zm(1:N),

where $z_{m}\left(1:N\right)$ denotes the first *N* elements of $z_{m}$. It is worth noting that $y_{mm}$ and ${y^{\prime }}_{mi}$ defined in (21) can be calculated by the Hadamard product and the FFT operations:

(32) ymm=λDfpmm=λwt∘pmm,y′mi=Re14N21−λFDtFHpmi=18N21−λFDtFH+FHDtFpmi=14N1−λFwt∘F−1pmi+F−1wt∘Fpmi.

From the derivation above, it is easy to see that the calculation of the phase gradient is a little bit cumbersome. In order to make the calculation process concise, we write the gradient in the form of matrix. Let $X=[x_{1},...,x_{M}]$ and $\Phi =[\phi_{1},...,\phi_{M}]$ denote the matrix of waveform set and the corresponding phase matrix, respectively. Then, the spectrum matrix of X is $S=F_{c}\left(X\right)=\left[f_{1},...,f_{M}\right]$. Define:

(33) $$\begin{array}{cc} Y & =\left[y_{11},...,y_{MM}\right]=\lambda \left(w_{f}1_{M}^{T}\right)\circ S\circ S^{*},\\ Y_{1} & =\left[\sum_{i=1}^{M}f_{i}\circ {y^{\prime }}_{1i},...,\sum_{i=1}^{M}f_{i}\circ {y^{\prime }}_{Mi}\right], \end{array}$$

then the matrix form of (29) is given by:

(34) $$Z=\left[z_{1},...,z_{M}\right]=F_{c}^{-1}\left(S\circ Y+Y_{1}\right).$$

Thus, according to (31), the matrix of phase gradient G can be expressed as:

(35) G=∇ϕ1JT,...,∇ϕMJT=8NRe−jX*∘Z(1:N,:),

where Z(1:N,:) denotes the submatrix formed with the first *N* rows of Z. By defining Y and $Y_{1}$, we can easily calculate the gradient matrix. However, it is still hard to calculate $Y_{1}$ directly by (33). To efficiently calculate $Y_{1}$, define:

(36) $$Y^{\prime }=\left[\begin{array}{ccc} {y^{\prime }}_{11} & \cdots & {y^{\prime }}_{1M}\\ \vdots & \ddots & \vdots \\ {y^{\prime }}_{M1} & \cdots & {y^{\prime }}_{MM} \end{array}\right]\in C^{2NM\times M},S_{rep}={\left[\begin{array}{c} S\\ \vdots \\ S \end{array}\right]}_{2NM\times M}.$$

Then it is easy to verify that:

(37) $$\left(S_{rep}\circ Y^{\prime }\right)1_{M}=\mathrm{vec}\left(Y_{1}\right),$$

where $\mathrm{vec}\left(Y_{1}\right)$ denotes the column vector consisting of all of the columns of $Y_{1}$. Thus, we can obtain $Y_{1}$ via the inverse operation of $\mathrm{vec}\left(\cdot \right)$, i.e.,

(38) $$Y_{1}=\mathrm{ve}c^{-1}\left(\left(S_{rep}\circ Y^{\prime }\right)1_{M}\right),$$

where $Y^{\prime }$ can be calculated by (32). It is easy to see that ${y^{\prime }}_{mi}$ can be implemented by four FFT (IFFT) operations. Since ${y^{\prime }}_{mi}={y^{\prime }}_{im}^{*}$, the calculation of $Y^{\prime }$ takes $4\frac{M(M+1)}{2}=2M^{2}+2M$ FFT (IFFT) operations.

### 3.2. Step Size Calculation via Taylor Series Expansion

The traditional methods for obtaining the step size are the linear search methods, which require many iterations and thus are quite time consuming. To reduce the computing expense, here we propose to calculate the step size directly. Assume that $\Phi^{l}$ is the phase matrix of the present iteration point $X^{l}=\left[x_{1}^{l},...,x_{M}^{l}\right]=e^{j\Phi^{l}}$, and $D^{l}=\left[d_{1}^{l},...,d_{M}^{l}\right]$ is the descent direction. Then, the new iteration point can be denoted as:

(39) $$\begin{array}{cc} \Phi^{l+1} & =\Phi^{l}+\mu D^{l},\\ X^{l+1} & =e^{j\Phi^{l+1}}=X^{l}\circ e^{j\mu D^{l}}, \end{array}$$

i.e.,

(40) $$x_{i}^{l+1}=x_{i}^{l}\circ e^{j\mu d_{i}^{l}},\;i=1,...,M,$$

where μ is the step size. Thus, the linear search problem can be formulated as the following minimization problem:

(41) $$\min_{\mu >0}\;\;h\left(\mu \right)=J_{T}\left(X^{l+1}\right)=\sum_{i=1}^{M}{\left(p_{ii}^{l+1}\right)}^{H}{\mathrm{Qp}}_{ii}^{l+1}+\sum_{i=1}^{M}\sum_{j=1}^{M}{\left(p_{ij}^{l+1}\right)}^{H}{Q^{\prime }p}_{ij}^{l+1}.$$

By taking the derivative of (41), we have:

(42) ∂h∂μ=2Re∑i=1M∂piil+1H∂μQpiil+1+∑i=1M∑j=1M∂pijl+1H∂μQ′pijl+1.

To simplify (41) and (42), we first deduce $p_{ij}^{l+1}$. By using the Taylor series expansion, $p_{ij}^{l+1}$ can be approximated as (see Appendix C):

(43) $$p_{ij}^{l+1}\approx f_{i}\circ f_{j}^{*}+j\left({f^{\prime }}_{i}\circ f_{j}^{*}-f_{i}\circ {f^{\prime }}_{j}^{*}\right)\mu -\frac{1}{2}\left({f^{\prime \prime }}_{i}\circ f_{j}^{*}+f_{i}\circ {f^{\prime \prime }}_{j}^{*}-2{f^{\prime }}_{i}\circ {f^{\prime }}_{j}^{*}\right)\mu^{2},$$

where:

(44) $$f_{i}=F\left(x_{i}^{l}\right),{f^{\prime }}_{i}=F\left(x_{i}^{l}\circ d_{i}^{l}\right),{f^{\prime \prime }}_{i}=F\left(x_{i}^{l}\circ d_{i}^{l}\circ d_{i}^{l}\right).$$

Let

(45) $$\begin{array}{cc} p_{ij}^{l} & =f_{i}\circ f_{j}^{*},\\ c_{ij} & =j\left({f^{\prime }}_{i}\circ f_{j}^{*}-f_{i}\circ {f^{\prime }}_{j}^{*}\right),\\ {c^{\prime }}_{ij} & =-\frac{1}{2}\left({f^{\prime \prime }}_{i}\circ f_{j}^{*}+f_{i}\circ {f^{\prime \prime }}_{j}^{*}-2{f^{\prime }}_{i}\circ {f^{\prime }}_{j}^{*}\right), \end{array}$$

then (43) can be rewritten as:

(46) $$p_{ij}^{l+1}\approx p_{ij}^{l}+c_{ij}\mu +{c^{\prime }}_{ij}\mu^{2}={\bar{p}}_{ij}^{l+1}.$$

By replacing $p_{ij}^{l+1}$ with ${\bar{p}}_{ij}^{l+1}$, the approximate function of h(μ) in (41) can be denoted as:

(47) $$h_{1}\left(\mu \right)=\sum_{i=1}^{M}{\left({\bar{p}}_{ii}^{l+1}\right)}^{H}{Q\bar{p}}_{ii}^{l+1}+\sum_{i=1}^{M}\sum_{j=1}^{M}{\left({\bar{p}}_{ij}^{l+1}\right)}^{H}{Q^{\prime }\bar{p}}_{ij}^{l+1}.$$

Since $p_{ij}^{l+1}={\bar{p}}_{ij}^{l+1}$ holds when μ=0, it is easy to verify that:

(48) $$h\left(0\right)=h_{1}\left(0\right),{\left.\frac{\partial h}{\partial \mu }\right|}_{\mu =0}={\left.\frac{\partial h_{1}}{\partial \mu }\right|}_{\mu =0},$$

which indicates that h(μ) and $h_{1}\left(\mu \right)$ have the same function value and slope at μ=0. As $D^{l}$ is the descent direction, the slopes of these two functions are less than zero. Thus, these two functions have at least one minimum point greater than zero. Since h(μ) is sensitive to the waveform phases, the optimal step size of h(μ) is very small and close to zero. Consequently, we can use the minimum point of $h_{1}\left(\mu \right)$ to approximate the optimal step size.

To calculate the minimum point of $h_{1}\left(\mu \right)$, we replace $p_{ij}^{l+1}$ in (42) with ${\bar{p}}_{ij}^{l+1}$, then the derivative of $h_{1}\left(\mu \right)$ with respective to μ is given by:

(49) ∂h1∂μ=2Re∑i=1MciiH+2c′iiHμQpiil+ciiμ+c′iiμ2+∑i=1M∑j=1McijH+2c′ijHμQ′pijl+cijμ+c′ijμ2=aμ3+bμ2+cμ+d,

where:

(50) a=2Re2∑i=1Mc′iiHQc′ii+2∑i=1M∑j=1Mc′ijHQ′c′ij,b=2Re∑i=1M2c′iiHQcii+ciiHQc′ii+∑i=1M∑j=1M2c′ijHQ′cij+cijHQ′c′ij,c=2Re∑i=1M2c′iiHQpiil+ciiHQcii+∑i=1M∑j=1M2c′ijHQ′pijl+cijHQ′cij,d=2Re∑i=1MciiHQpiil+∑i=1M∑j=1McijHQ′pijl.

To simplify the calculation, let:

(51) $$\begin{array}{cc} h_{ij} & =F^{-1}\left(c_{ij}\right)=\frac{1}{2N}F^{H}c_{ij},\\ {h^{\prime }}_{ij} & =F^{-1}\left({c^{\prime }}_{ij}\right)=\frac{1}{2N}F^{H}{c^{\prime }}_{ij}. \end{array}$$

Then (50) can be rewritten as:

(52) a=4ReλwfT∑i=1Mc′ii*∘c′ii+1−λwtT∑i=1M∑j=1Mh′ij*∘h′ij,b=2ReλwfT∑i=1M2c′ii*∘cii+cii*∘c′ii+1−λwtT∑i=1M∑j=1M2h′ij*∘hij+hij*∘h′ij,c=2ReλwfT∑i=1M2c′ii*∘piil+cii*∘cii+1−λwtT∑i=1M∑j=1M2h′ij*∘rijl+hij*∘hij,d=2ReλwfT∑i=1Mcii*∘piil+1−λwtT∑i=1M∑j=1Mhij*∘rijl,

where the first term of each equality follows from the fact $a^{H}\mathrm{Qb}=\lambda a^{H}D_{f}b=w_{f}^{T}\left(a^{*}\circ b\right)$ (a,b are the arbitrary vectors), and the second term of each equality follows from the fact $a^{H}Q^{\prime }b=\frac{1}{4N^{2}}\left(1-\lambda \right)a^{H}FD_{t}F^{H}b=\left(1-\lambda \right)w_{t}^{T}\left({\left(F^{-1}\left(a\right)\right)}^{*}\circ F^{-1}\left(b\right)\right)$. Let $\frac{\partial h_{1}}{\partial \mu }=0$, then the minimum point of $h_{1}\left(\mu \right)$ can be obtained by solving the following cubic equation:

(53) $$a\mu^{3}+b\mu^{2}+c\mu +d=0$$

It is well known that a cubic equation with real coefficients has three roots, in which there is at least a real root. Therefore, we can choose the positive root that is closest to zero as the precise estimation of the step size.

In order to facilitate the calculation, we write the above derivation in the form of matrix. Define:

(54) $$S=\left[f_{1},...,f_{M}\right],S^{\prime }=\left[{f^{\prime }}_{1},...,{f^{\prime }}_{M}\right],\;S^{\prime \prime }=\left[{f^{\prime \prime }}_{1},...,{f^{\prime \prime }}_{M}\right],$$

then according to (44) we have:

(55) $$S=F_{c}\left(X^{l}\right),S^{\prime }=F_{c}\left(X^{l}\circ D^{l}\right),S^{\prime \prime }=F_{c}\left(X^{l}\circ D^{l}\circ D^{l}\right).$$

Let:

(56) $$\begin{array}{cc} P & =\left[p_{11}^{l},...,p_{M1}^{l},p_{12}^{l},...,p_{M2}^{l},......,p_{1M}^{l},...,p_{MM}^{l}\right],\\ C & =\left[c_{11},...,c_{M1},c_{12},...,c_{M2},......,c_{1M},...,c_{MM}\right],\\ C^{\prime } & =\left[{c^{\prime }}_{11},...,{c^{\prime }}_{M1},{c^{\prime }}_{12},...,{c^{\prime }}_{M2},......,{c^{\prime }}_{1M},...,{c^{\prime }}_{MM}\right],\\ P_{d} & =\left[p_{11}^{l},p_{22}^{l},...,p_{MM}^{l}\right],\\ C_{d} & =\left[c_{11},c_{22},...,c_{MM}\right],\\ {C^{\prime }}_{d} & =\left[{c^{\prime }}_{11},{c^{\prime }}_{22},...,{c^{\prime }}_{MM}\right], \end{array}$$

Then the inverse Fourier transforms of $P,C,C^{\prime }$ can be denoted as:

(57) $$R=F_{c}^{-1}\left(P\right),H=F_{c}^{-1}\left(C\right),H^{\prime }=F_{c}^{-1}\left(C^{\prime }\right).$$

According to (52), (56) and (57), the coefficients of the cubic equation can be reformulated as:

(58) a=4ReλwfTC′d*∘C′d1M+1−λwtTH′*∘H′1M2,b=2ReλwfT2C′d*∘Cd+Cd*∘C′d1M+1−λwtT2H′*∘H+H*∘H′1M2,c=2ReλwfT2C′d*∘Pd+Cd*∘Cd1M+1−λwtT2H′*∘R+H*∘H1M2,d=2ReλwfTCd*∘Pd1M+1−λwtTH*∘R1M2.

### 3.3. Algorithm Summary

After deducing the phase gradient and the step size, it is easy to solve the unconstrained problem (17) by using the conjugate gradient algorithm (CGA). Here, we apply the classical Polak–Ribiere–Polyak CGA (PRP-CGA) to deal with the waveform design problem. The searching direction of the PRP-CGA can be expressed as:

(59) $$d^{l+1}=-g^{l+1}+\frac{{\left(g^{l+1}-g^{l}\right)}^{T}g^{l+1}}{{∥g^{l}∥}^{2}}d^{l},$$

where $g^{l}$ and $d^{l}$ are the gradient vector and the direction vector, respectively. For the problem here, $g^{l}$ and $d^{l}$ are defined as:

(60) $$g^{l}={\left[{\left(g_{1}^{l}\right)}^{T},...,{\left(g_{M}^{l}\right)}^{T}\right]}^{T},d^{l}={\left[{\left(d_{1}^{l}\right)}^{T},...,{\left(d_{M}^{l}\right)}^{T}\right]}^{T},$$

where $g_{m}^{l}=\nabla_{\phi_{m}}J_{T}\left(X^{l}\right),m=1,...,M$. Since the step size is an approximate value, ${\left(g^{l+1}\right)}^{T}d^{l}$ is not equal to zero. Thus, $d^{l+1}$ may not be descendent, i.e.,

(61) $${\left(g^{l+1}\right)}^{T}d^{l+1}=-{∥g^{l+1}∥}^{2}+\frac{{\left(g^{l+1}-g^{l}\right)}^{T}g^{l+1}}{{∥g^{l}∥}^{2}}{\left(g^{l+1}\right)}^{T}d^{l}<0$$

is not always satisfied. For guaranteeing that the searching direction is descendant, we adopt the following modified direction:

(62) $$\begin{array}{cc} {\tilde{d}}^{l+1} & =-g^{l+1}+\frac{{\left(g^{l+1}-g^{l}\right)}^{T}g^{l+1}}{{∥g^{l}∥}^{2}}d^{l},\\ d^{l+1} & =\left\{\begin{array}{cc} {\tilde{d}}^{l+1}, & {\left(g^{l+1}\right)}^{T}{\tilde{d}}^{l+1}<0\\ -g^{l+1}, & {\left(g^{l+1}\right)}^{T}{\tilde{d}}^{l+1}\geq 0 \end{array}\right.. \end{array}$$

Actually, (59) can also be expressed as:

(63) $$D^{l+1}=-G^{l+1}+\frac{{\left(g^{l+1}-g^{l}\right)}^{T}g^{l+1}}{{∥g^{l}∥}^{2}}D^{l}.$$

Since

(64) $$\begin{array}{cc} {∥g^{l}∥}^{2} & =\sum_{m=1}^{M}{∥g_{m}^{l}∥}^{2}={∥G^{l}∥}_{F}^{2},\\ {\left(g^{l+1}-g^{l}\right)}^{T}g^{l+1} & ={∥G^{l+1}∥}_{F}^{2}-1_{N}^{T}\left(G^{l+1}\circ G^{l}\right)1_{M}, \end{array}$$

and (63) can be rewritten as:

(65) $$D^{l+1}=-G^{l+1}+\frac{{∥G^{l+1}∥}_{F}^{2}-1_{N}^{T}\left(G^{l+1}\circ G^{l}\right)1_{M}}{{∥G^{l}∥}_{F}^{2}}D^{l}.$$

Thus, (62) can be expressed as the following matrix form:

(66) $$\begin{array}{cc} {\tilde{D}}^{l+1} & =-G^{l+1}+\frac{{∥G^{l+1}∥}_{F}^{2}-1_{N}^{T}\left(G^{l+1}\circ G^{l}\right)1_{M}}{{∥G^{l}∥}_{F}^{2}}D^{l},\\ D^{l+1} & =\left\{\begin{array}{c} {\tilde{D}}^{l+1},1_{N}^{T}\left(G^{l+1}\circ {\tilde{D}}^{l+1}\right)1_{M}<0\\ -G^{l+1},1_{N}^{T}\left(G^{l+1}\circ {\tilde{D}}^{l+1}\right)1_{M}\geq 0 \end{array}\right.. \end{array}$$

On the basis of the above derivation, the gradient-based algorithm, which we call Gra-WeCorr-SFW, is summarized in Algorithm 1.

## 4. Simplified Algorithm for the Design Problem without Considering the Correlation Weights

In Section 3, we present a gradient-based algorithm to handle Problem (17). From (9), we can see that the criterion $J_{CF}$ can be simplified when $w_{t}\left(k\right)=1,k=1-N,...,N-1$ (i.e., the correlation weights are not taken into account). Thus, in this section, we derive a simplified algorithm for the design problem without considering the correlation weights. Let $w_{t}\left(k\right)=1,k=1-N,...,N-1$, then the criterion $J_{CF}$ can be simplified as:

(67) $$J_{CF}=\frac{1}{2N}\sum_{i=1}^{M}\sum_{j=1}^{M}p_{ij}^{H}p_{ij}=\frac{1}{2N}\sum_{i=1}^{M}\sum_{j=1}^{M}p_{ii}^{H}p_{jj}=\frac{1}{2N}\sum_{i=1}^{M}p_{ii}^{H}\sum_{j=1}^{M}p_{jj}.$$

Thus we can rewrite the problem (17) as:

(68) $$J_{T}=\lambda \sum_{i=1}^{M}p_{ii}^{H}D_{f}p_{ii}+\lambda_{1}\sum_{i=1}^{M}p_{ii}^{H}\sum_{j=1}^{M}p_{jj},$$

where $\lambda_{1}=\frac{1-\lambda }{2N}$. Like the derivation in Section 3, we deduce the gradient and the step size.

**Algorithm 1:** Gra-WeCorr-SFW.**Initialization**: $l=0,\lambda ,M,N,w_{t},w_{f},X^{0}=\left[x_{1}^{0},...,x_{M}^{0}\right],$       $S=F_{c}\left(X^{0}\right),G^{0},D^{0}=-G^{0}.$
**Repeat**
1: $S^{\prime }=F_{c}\left(X^{l}\circ D^{l}\right),S^{\prime \prime }=F_{c}\left(X^{l}\circ D^{l}\circ D^{l}\right).$2: Compute $P,C,C^{\prime },P_{d},C_{d},{C^{\prime }}_{d}$ according to (45).3: $R=F_{c}^{-1}\left(P\right),H=F_{c}^{-1}\left(C\right),H^{\prime }=F_{c}^{-1}\left(C^{\prime }\right).$4: Compute coefficients a,b,c,d according to (58).5: Solve the cubic Equation (53), and then choose the positive root that is closest to zero as the step size $\mu^{l}$.6: $X^{l+1}=X^{l}\circ e^{j\mu^{l}D^{l}},S=F_{c}\left(X^{l+1}\right).$7: $Y=\lambda \left(w_{f}1_{M}^{T}\right)\circ S\circ S^{*}.$8: Compute $Y^{\prime }$ according to (32).9: $S_{rep}={\left[S^{T},...,S^{T}\right]}_{2NM\times M}^{T},Y_{1}=\mathrm{ve}c^{-1}\left(\left(S_{rep}\circ Y^{\prime }\right)1_{M}\right).$10: $Z=F_{c}^{-1}\left(S\circ Y+Y_{1}\right).$11: Gl+1=8NRe−jXl+1*∘Z(1:N,:).12: Compute the searching direction $D^{l+1}$ according to (66).13: l=l+1.**Until** convergence

### 4.1. Phase Gradient

According to (68), we deduce the derivative $\partial J_{T}/\partial \phi_{mn}$ as:

(69) ∂JT∂ϕmn=2Re∂pmmH∂ϕmnλwf∘pmm+λ1∑j=1Mpjj.

Let:

(70) $${y^{\prime \prime }}_{m}=\lambda w_{f}\circ p_{mm}+\lambda_{1}\sum_{j=1}^{M}p_{jj},$$

then according to (23), (69) can be rewritten as:

(71) ∂JT∂ϕmn=2Re∑k=12N∂pmm*(k)∂ϕmny′′m(k)=4Re−jxmm*∑k=12Nfm(k)y′′m(k)ej2π2Nnk.

By stacking (71) in a vector, the gradient is given by:

(72) ∇ϕmJT=8NRe−jxm*∘z′m1:N,

where ${z^{\prime }}_{m}=F^{-1}\left(f_{m}\circ {y^{\prime \prime }}_{m}\right)$. Define $Y_{2}=\left[{y^{\prime \prime }}_{1},...,{y^{\prime \prime }}_{M}\right],Z_{1}=\left[{z^{\prime }}_{1},...,{z^{\prime }}_{M}\right]$, then it is easy to verify that:

(73) $$\begin{array}{cc} Y_{2} & =\lambda \left(w_{f}1_{M}^{T}\right)\circ S\circ S^{*}+\lambda_{1}\left(S\circ S^{*}\right)1_{M}1_{M}^{T},\\ Z_{1} & =F_{c}^{-1}\left(S\circ Y_{2}\right). \end{array}$$

Thus, the gradient matrix can be expressed as:

(74) G=8NRe−jX*∘Z11:N,:.

### 4.2. Step Size

Similarly to (41), the linear search problem here can be denoted as:

(75) $$\min_{\mu >0}\;h\left(\mu \right)=\lambda \sum_{i=1}^{M}{\left(p_{ii}^{l+1}\right)}^{H}D_{f}p_{ii}^{l+1}+\lambda_{1}\sum_{i=1}^{M}{\left(p_{ii}^{l+1}\right)}^{H}\sum_{j=1}^{M}p_{jj}^{l+1}.$$

By taking the derivative of h(μ), we have:

(76) ∂h∂μ=2Reλ∑i=1M∂piil+1∂μHDfpiil+1+λ1∑i=1M∂piil+1∂μH∑j=1Mpjjl+1.

Then, the approximate derivative can be obtained by replacing $p_{pq}^{l+1}$ with ${\bar{p}}_{pq}^{l+1}$ defined in (46):

(77) ∂h∂μ≈2Reλ∑i=1Mcii+2c′iiμHDfpiil+ciiμ+c′iiμ2+λ1∑i=1Mcii+2c′iiμH∑j=1Mpjjl+cjjμ+c′jjμ2.

According the definition (56), we have:

(78) $$\sum_{i=1}^{M}p_{ii}^{l}=P_{d}1_{M},\sum_{i=1}^{M}c_{ii}=C_{d}1_{M},\sum_{i=1}^{M}{c^{\prime }}_{ii}={C^{\prime }}_{d}1_{M},$$

where $P_{d}\;,C_{d}$ and ${C^{\prime }}_{d}$ can be expressed as the following equalities according to (45):

(79) Pd=S∘S*,Cd=−2ImS′∘S*,C′d=−ReS′′∘S*−S′∘S′*.

Thus, (77) can be written more compactly as:

(80) $$\frac{\partial h}{\partial \mu }\approx a_{1}\mu^{3}+b_{1}\mu^{2}+c_{1}\mu +d_{1},$$

where:

(81) a1=4ReλwfTC′d*∘C′d1M+λ1C′d1MHC′d1M,b1=2ReλwfT2C′d*∘Cd+Cd*∘C′d1M+λ12C′d1MHCd1M+Cd1MHC′d1M,c1=2ReλwfT2C′d*∘Pd+Cd*∘Cd1M+λ12C′d1MHPd1M+Cd1MHCd1M,d1=2ReλwfTCd*∘Pd1M+λ1Cd1MHPd1M.

By solving the cubic equation $a_{1}\mu^{3}+b_{1}\mu^{2}+c_{1}\mu +d_{1}=0$, the step size can be easily obtained.

### 4.3. Algorithm Summary

In the previous two subsections, the gradient and step size for Problem (68) are derived. Then, the simplified algorithm (which we call Gra-Corr-SFW) for the design problem without considering the correlation weights is summarized in Algorithm 2. It is easy to observe that both Algorithm 1 and Algorithm 2 can be easily implemented by the (I)FFT operations and the Hadamard product. Since the Hadamard product is more efficient than the (I)FFT operation, we mainly use the number of (I)FFT operations to measure the time complexity of the algorithms. From Section 3, we know that the calculation of $Y^{\prime }$ needs $2M^{2}+2M$ (I)FFT operations. Thus, the Gra-WeCorr-SFW requires $5M^{2}+6M$ (I)FFT operations at each iteration. Compared to the Gra-WeCorr-SFW, the Gra-Corr-SFW is simpler and only needs 4M (I)FFT operations at each iteration. It is worth noting that the calculation of the cubic function is very simple and needs only a small amount of computation.

**Algorithm 2:** Gra-Corr-SFW.**Initialization**: $l=0,\lambda ,\lambda_{1},M,N,w_{t},w_{f},X^{0}=\left[x_{1}^{0},...,x_{M}^{0}\right],$       $S=F_{c}\left(X^{0}\right),G^{0},D^{0}=-G^{0}.$
**Repeat**
1: $S^{\prime }=F_{c}\left(X^{l}\circ D^{l}\right),S^{\prime \prime }=F_{c}\left(X^{l}\circ D^{l}\circ D^{l}\right).$2: Pd=S∘S*,Cd=-2ImS′∘S*,C′d=-ReS′′∘S*-S′∘S′*.3: Compute the coefficients $a_{1},b_{1},c_{1},d_{1}$ according to (81).4: Solve the cubic Equation (80), and then choose the positive root which is closest to zero as the step size $\mu^{l}$.5: $X^{l+1}=X^{l}\circ e^{j\mu^{l}D^{l}},S=F_{c}\left(X^{l+1}\right)$6: $Y_{2}=\lambda \left(w_{f}1_{M}^{T}\right)\circ S\circ S^{*}+\lambda_{1}\left(S\circ S^{*}\right)1_{M}1_{M}^{T}.$7: $Z_{1}=F_{c}^{-1}\left(S\circ Y_{2}\right).$8: Gl+1=8NRe-jXl+1*∘Z11:N,:.9: Compute the searching direction $D^{l+1}$ according to (66).10: l=l+1.**Until** convergence

To analyze the convergence speed, Table 1 presents the per iteration computational complexities of the proposed and existing algorithms. As shown in Table 1, the proposed Gra-WeCorr-SFW requires fewer (I)FFT operations than the MM-WeCorr-acc (MM-WeCorr-acceleration) at each iteration. Compared to these two algorithms, the per iteration computational complexities of the rest of the three algorithms (MM-Corr-acc (MM-Corr-acceleration), MDISAA-SFW and Gra-Corr-SFW), which do not consider the correlation weights, are much smaller. In addition to the per iteration computational complexity, the iteration number is also an important factor affecting the convergence speed. Thus, it is difficult to compare the convergence performance of the algorithms via the per iteration computational complexity. In the following section, several numerical experiments are provided to show the convergence performance of the proposed algorithms.

## 5. Numerical Experiments

To illustrate the effectiveness and superiority of the proposed algorithms, several numerical experiments are presented in this section. We first validate the monotonicity of the proposed algorithms and then assess the performance of the algorithms by designing three different waveform sets. The proposed algorithms are compared with the MM-Corr-acc [14], MM-WeCorr-acc [14] and MDISAA-SFW [37] algorithms, where MM-Corr-acc and MM-WeCorr-acc are the state-of-the-art algorithms for designing waveform sets with a good correlation property, and MDISAA-SFW is the state-of-the-art algorithm for designing the orthogonal waveform set (OWS) with the stopband constraint.

All of the experiments are performed on a PC with a 3.60-GHz i7-4790 CPU and 8GB RAM. The software environment is MATLAB 2012b. In the following experiments, all of the algorithms are initialized by the unimodular waveform sets with random phases.

### 5.1. Verification of The Monotonicity

In this subsection, we investigate the monotonicity of the proposed algorithms in three different waveform design problems, which are respectively complementary sets of sequences (CSS) design, waveform set design with zero correlation zone and orthogonal waveform set (OWS) design with the stopband constraint. In order to measure the monotonicity, define the relative error of the *l*-th iteration as follows:

(82) $$\varepsilon_{r}^{l}=\frac{h\left(\mu^{l}\right)-h\left({\dot{\mu }}^{l}\right)}{h\left(0\right)-h\left({\dot{\mu }}^{l}\right)},$$

where $\mu^{l}$ denotes the approximate step size of the *l*-th iteration, which is obtained via the Taylor series expansion, and ${\dot{\mu }}^{l}$ denotes the optimal step size obtained by the searching method. The smaller the value of $\varepsilon_{r}^{l}$ is, the closer the approximate step size is to the optimal step size. Thus, $\varepsilon_{r}^{l}$ can be used to express the accuracy of the approximate step size. When $\varepsilon_{r}^{l}<1$, we have $h\left(\mu^{l}\right)<h\left(0\right)$, which means the objective function is decreasing at the *l*-th iteration. Therefore, we can measure the monotonicity of the algorithms by using the following peak relative error $P_{re}$:

(83) $$P_{re}=\max\left(\varepsilon_{r}^{l},l=1,...,N_{I}\right),$$

where $N_{I}$ is the iteration number.

**Figure 1:**
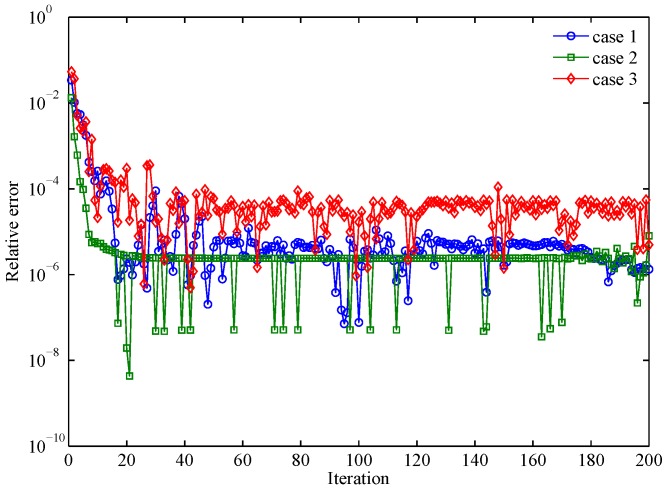
Evolution of the relative error with respect to the iteration number.

**Figure 2:**
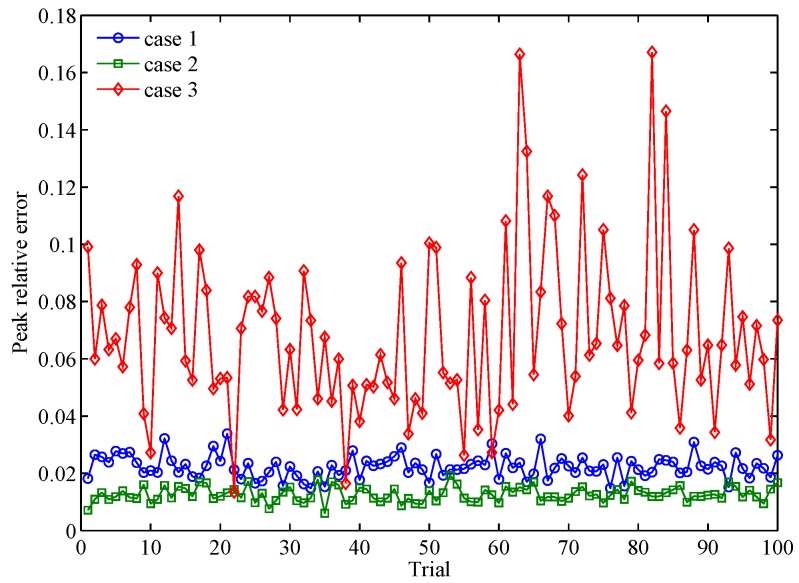
The peak relative errors of 100 random trials.

To simulate $\varepsilon_{r}^{l}$ and $P_{re}$, we use the Gra-WeCorr-SFW to design waveform sets with M=3 waveforms and each waveform of length N=256. The simulation parameters of different design problems are shown in Table 2. Additionally, we stop the algorithm after 200 iterations. Figure 1 shows the evolution curves of the relative error with respect to the iteration number. From Figure 1, we can see that the relative error is a little larger at the initial iterations. However, it decreases rapidly and is substantially below $10^{-4}$ after several iterations. In the whole iteration process, the relative error is less than one, which means that the objective function is monotonically decreasing. Figure 2 shows the peak relative error of 100 random trials. It is easy to see that the peak relative error of all three cases is very small and less than one. This indicates that the monotonicity of the Gra-WeCorr-SFW is not affected by the initial iteration point. Since the Gra-Corr-SFW is a simplified algorithm of the Gra-WeCorr-SFW, it can also guarantee that the objective function is monotonically decreasing at each iteration.

### 5.2. CSS Design or OWS Design

**Figure 3:**
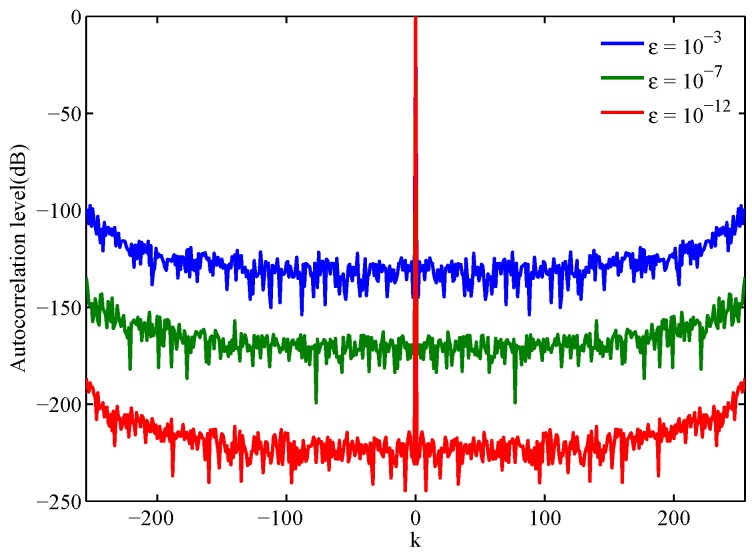
The autocorrelation sum of the waveform sets designed by the Gra-Corr-SFW with different ε. (N=256,M=3)

In Appendix A, we have demonstrated that the CSS design is equivalent to the OWS design. In this subsection, we apply the Gra-Corr-SFW to design CSS (or OWS). Since the lower bound of the CISL is zero, we choose CISL≤ε as the stopping criterion for all of the algorithms in this experiment. The weight coefficient λ and the correlation weights are the same as Case 1 in Table 2. Figure 3 shows the normalized autocorrelation sum of the waveform sets designed by the Gra-Corr-SFW, where the normalized autocorrelation sum is defined as:

(84) $$r_{sum}\left(k\right)=20\log_{10}\frac{\left|\sum_{m=1}^{M}r_{mm}\left(k\right)\right|}{\sum_{m=1}^{M}r_{mm}\left(0\right)},k=1-N,...,N-1.$$

From Figure 3, we can see that the autocorrelation sum of the CSS is a Delta function. Additionally, with the decrease of ε, the sidelobes of the CSS are getting lower and lower.

Next, we compare the performance between the proposed Gra-Corr-SFW and the state-of-the-art MM-Corr-acc by designing waveform sets with different waveform number *M* and waveform length *N*. For both algorithms, we choose *M* and *N* as follows:

(85) M=3k,N=256k,k=1,...,5.

**Figure 4:**
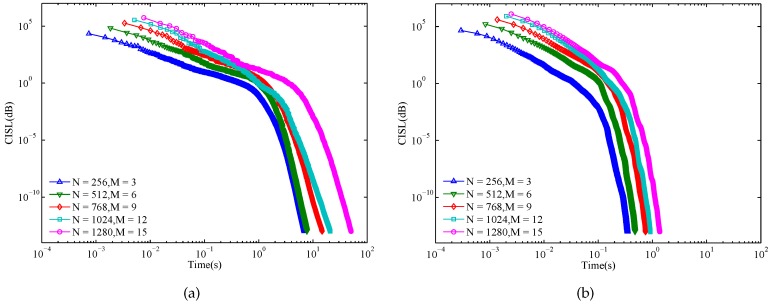
Evolution of the CISL with respect to the running time: (**a**) MM-Corr-acc; (**b**) Gra-Corr-SFW. $\left(\varepsilon =10^{-13}\right)$.

The evolution curves of MM-Corr-acc and Gra-Corr-SFW are respectively shown in Figure 4a,b. From these two subfigures, we observe that the convergence speed of the proposed Gra-Corr-SFW is much faster than that of the MM-Corr-acc. Even for (N=1280,M=15), the proposed algorithm takes 1.35 s to coverage to $\mathrm{CISL}\leq 10^{-13}$, while the MM-Corr-acc takes 48.11 s.

Further, to eliminate the randomness, we repeat the algorithms 100 times for each (M,N) pair and record the average iteration number $N_{I}$, the average running time *t* and the average peak sidelobe level of the autocorrelation sum $P_{sum}$, where $P_{sum}$ is defined as:

(86) $$P_{sum}=\max\left(r_{sum}\left(k\right),k=1,...,N-1\right).$$

Then, we choose $\varepsilon =10^{-1}$ for the stopping criterion. The performance parameters $\left(N_{I},t,P_{sum}\right)$ of these two algorithms are provided in Table 3. As can be seen from this Table, since the algorithms stop the iteration when $\mathrm{CISL}\leq 10^{-1}$, the $P_{sum}$ of these two algorithms are basically the same. At the same time, the average running time in Table 3 shows that the Gra-Corr-SFW is an order of magnitude faster than the MM-Corr-acc. From Table 1, we can see that the per iteration computational complexities of both the MM-Corr-acc and the Gra-Corr-SFW are O(8MNlog2N). Therefore, the reason for the slower convergence of the MM-Corr-acc may be that the MM strategy in this algorithm makes the objective function loose, so that the iteration number of the MM-Corr-acc is larger than that of the Gra-Corr-SFW (as shown in Table 3).

### 5.3. Waveform Set Design with Zero Correlation Zone

**Figure 5:**
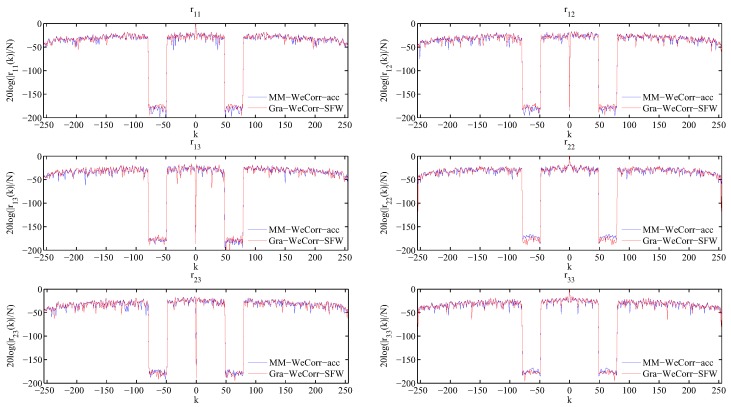
The normalized auto- and cross-correlations of the waveform sets designed by MM-WeCorr-acc and Gra-WeCorr-SFW. $\left(\varepsilon =10^{-10}\right)$.

To verify the effectiveness of the Gra-WeCorr-SFW, we consider the problem of suppressing the correlation sidelobes at the specified intervals, i.e., designing the waveform set with the zero correlation zone (ZCZ), and compare the performance with the MM-WeCorr-acc. The experimental parameters are the same as Case 2 in Table 2. For both algorithms, we choose the value of the objective function ψ in (4) as the stopping criterion, i.e., ψ≤ε. The auto- and cross-correlations of the waveform sets designed by MM-WeCorr-acc and Gra-WeCorr-SFW are shown in Figure 5. From this figure, we can see that the proposed algorithm can also generate a waveform set with correlation sidelobes that are almost zero at the specified intervals.

**Figure 6:**
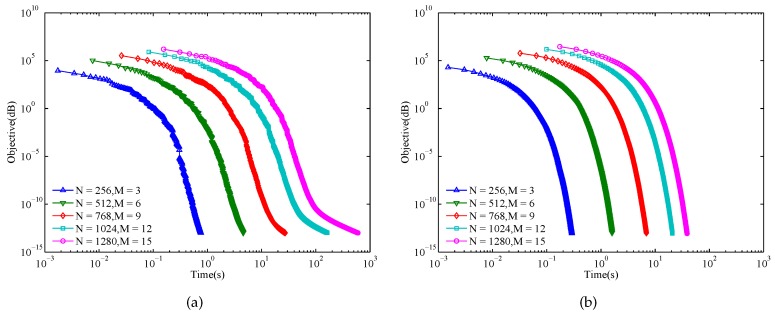
Evolution of the objective function with respect to the running time: (**a**) MM-WeCorr-acc; (**b**) Gra-WeCorr-SFW $\left(\varepsilon =10^{-13}\right)$.

Similar to Figure 4, for each (M,N) pair, we simulate the evolution curves of the objective function ψ with respect to the running time. Additionally, the results of these two algorithms are presented in Figure 6. It is easy to see that the Gra-WeCorr-SFW is faster than the MM-WeCorr-acc. In addition, we can also find that when the objective function ψ is less than $10^{-11}$, the convergence speed of the MM-WeCorr-acc decreases, especially for a large (M,N) pair.

Further, Table 4 presents the comparison of performance parameters between MM-WeCorr-acc and Gra-WeCorr-SFW, where $P_{zcz}$ is the peak sidelobe level at the zero correlation zone defined as:

(87) $$P_{zcz}=\max\left(20\log_{10}\left|\frac{r_{ij}\left(k\right)}{N}\right|,i,j=1,...,M,k\in \left[51,80\right]\right).$$

Similarly, for each (M,N) pair, the algorithms are repeated 100 times. As can be seen from Table 4, the $P_{zcz}$ of the Gra-WeCorr-SFW is a little bit lower than that of the MM-WeCorr-acc. Moreover, due to fewer iterations, the average running time of the Gra-WeCorr-SFW is about 50% of that of the MM-WeCorr-acc, which indicates that the former is more computationally efficient.

### 5.4. OWS Design with the Stopband Constraint

**Figure 7:**
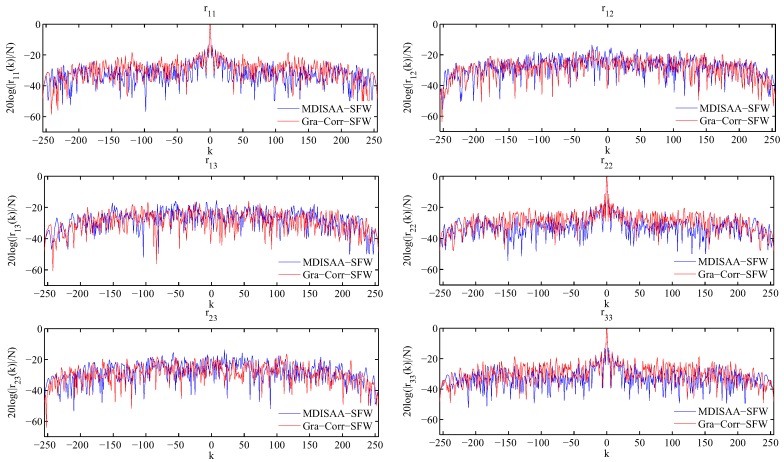
The correlation levels of the waveform sets designed by MDISAA-SFW and Gra-Corr-SFW.

**Figure 8:**
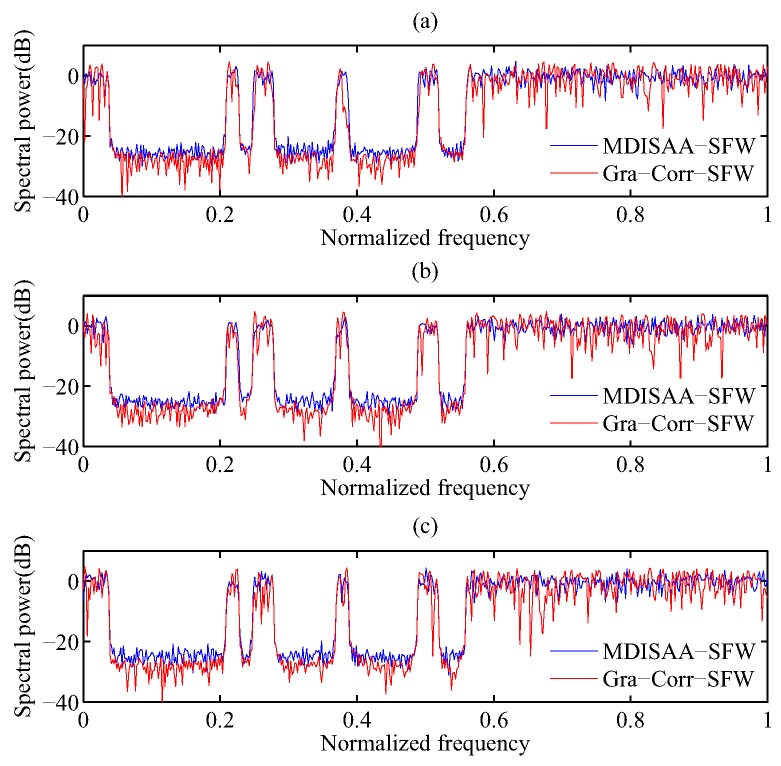
The spectral power of the waveform sets designed by MDISAA-SFW and Gra-Corr-SFW.

In this subsection, we consider the problem of designing OWS with the stopband constraint. Since the Gra-Corr-SFW is a simplified version of the Gra-WeCorr-SFW and needs much fewer (I)FFT operations, it is more efficient than the Gra-WeCorr-SFW. Thus, we only investigate the performance of the Gra-Corr-SFW and compare it with the MDISAA-SFW. Here, ${∥X^{l+1}-X^{l}∥}_{F}\leq 10^{-4}$ is employed as the stopping criterion, and the experimental parameters are the same as Case 3 in Table 2. First, we apply these two algorithms to design OWS with the stopband constraint. Suppose the waveform number *M* is three and the waveform length *N* is 256. The correlation levels and spectral power of the waveform sets designed by MDISAA-SFW and Gra-Corr-SFW are shown in Figure 7 and Figure 8. From Figure 7, we can see that the waveform set designed by the MDISAA-SFW has better autocorrelation performance, while the waveform set designed by the Gra-Corr-SFW has better cross-correlation performance. This is because the MDISAA-SFW optimizes the autocorrelation explicitly and, thus, places more emphasis on suppressing the autocorrelation sidelobes. Figure 8 indicates that the stopband spectral power of the waveform set designed by the Gra-Corr-SFW is less than that of the waveform set designed by the MDISAA-SFW.

In order to fully compare the algorithms, we define the peak autocorrelation sidelobe $P_{ac}$, peak cross-correlation $P_{cc}$, peak stopband power $P_{sp}$ and integrated sidelobe level (ISL) [16] as follows:

(88) Pac=max20log10rii(k)N,i=1,...,M,k=2,...,N−1,Pcc=max20log10rij(k)N,i,j=1,...,M,i≠j,k=0,...,N−1,Psp=maxPstopi,i=1,...,M,ISL=∑i=1M∑k=−N+1k≠0N−1rii(k)2+∑i=1M∑j=1j≠iM∑k=−N+1N−1rij(k)2,

where $P_{stop}^{i}$ denotes the peak stopband power of the waveform $x_{i}$. In the above four performance parameters, $P_{ac}$ and $P_{cc}$ are used to measure the autocorrelation and cross-correlation performances, respectively; $P_{sp}$ is the parameter related to the stopband performance; and ISL indicates the overall sidelobe performance. Since the decrease of the bandwidth leads to the broadening of the main lobe, $r_{ii}\left(1\right),i=1,...,M$ can be regarded as the autocorrelation main lobe. Thus, the definition of $P_{ac}$ does not take k=1 into account. Here, we consider the waveform sets with M=3 waveforms and each waveform of length N∈{256,512,1024}. For each (M,N) pair, we repeat these two algorithms 100 times and record the average values of the performance parameters. The comparison of the performance parameters between MDISAA-SFW and Gra-Corr-SFW is shown in Table 5. In addition to the parameters defined in (88), the average iteration number $N_{I}$ and the running time *t* are provided in Table 5. From this table, we obtain three findings, as follows. Above all, the $P_{cc}$ and $P_{sp}$ of the Gra-Corr-SFW are lower than that of the MDISAA-SFW, which means that the cross-correlation and stopband performances of the Gra-Corr-SFW are better than that of the MDISAA-SFW. However, in terms of autocorrelation performance, the Gra-Corr-SFW is inferior to the MDISAA-SFW. From the ISL in Table 5, it is easy to see that the overall sidelobe performance of the Gra-Corr-SFW is better than that of the MDISAA-SFW. Furthermore, although the per iteration computational complexity of the Gra-Corr-SFW is slightly higher than that of the MDISAA-SFW (see Table 1), the Gra-Corr-SFW needs fewer iterations due to the fast convergence of the gradient algorithm, which makes the proposed algorithm more efficient. Finally, with the increase of the waveform length, both the correlation and stopband performances of these two algorithms are improved, especially the cross-correlation performance.

**Figure 9:**
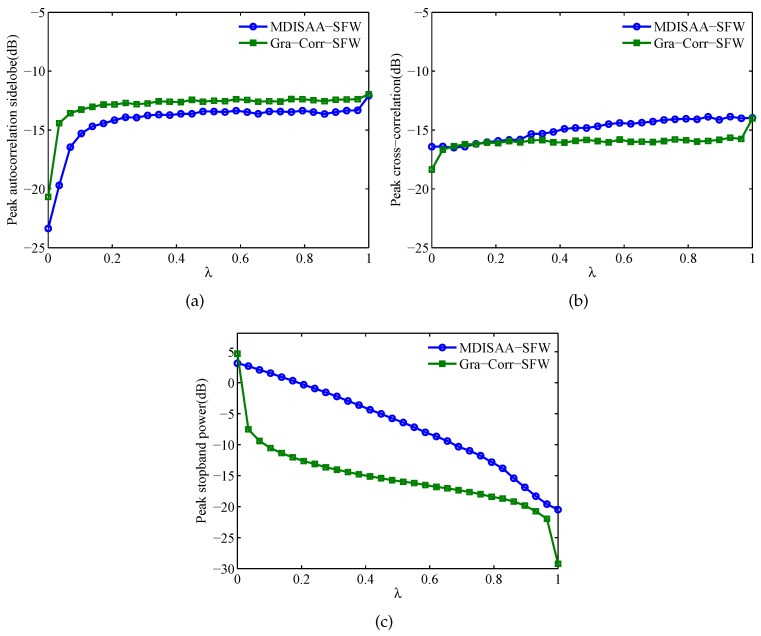
The comparison of $P_{ac}$, $P_{cc}$ and $P_{sp}$ between MDISAA-SFW and Gra-Corr-SFW versus λ from 0–1. (**a**) $P_{ac}$; (**b**) $P_{cc}$; (**c**) $P_{sp}$ (M=3,N=256).

Next, we investigate the performance of the algorithms under different λ. The average values (100 trials) of $P_{ac}$, $P_{cc}$ and $P_{sp}$ are shown in Figure 9. From this figure, we can see that with the increase of λ, the $P_{ac}$ and $P_{cc}$ of these two algorithms change slowly, while the $P_{sp}$ is sensitive to the change of λ. It can also be observed that except the autocorrelation performance, the cross-correlation and stopband performances of the Gra-Corr-SFW are better than that of the MDISAA-SFW. This means that the stopband property can be easily obtained by using the Gra-Corr-SFW. Moreover, since the correlation performance changes slowly with the increase of λ, we can choose a relatively large λ (e.g., λ∈(0.85,0.95)) for the Gra-Corr-SFW to obtain both good correlation and stopband properties.

## 6. Conclusions

In this paper, we propose an efficient algorithm named Gra-WeCorr-SFW for designing the waveform set with a good correlation and stopband properties. The algorithm optimizes the objective function directly and can guarantee that the objective function is monotonically decreasing at each iteration. By changing the design parameters, the Gra-WeCorr-SFW can be used to generate different waveform sets, such as CSS, the waveform set with ZCZ and OWS with the stopband constraint. As the main steps can be implemented by the FFT operations and the Hadamard product, the proposed algorithm is computationally efficient and can be used to design waveform sets with large *M* and *N*. Besides, the simplified version of the Gra-WeCorr-SFW (named Gra-Corr-SFW) is proposed for the design problem without considering the correlation weights. Compared to the Gra-WeCorr-SFW, the simplified algorithm requires fewer FFT operations and is faster. Numerical experiments show that the proposed algorithms are faster than the state-of-the-art algorithms (MM-WeCorr-acc and MM-Corr-acc) when designing CSS or the waveform set with ZCZ. In the case of designing OWS with the stopband constraint, the simplified algorithm has better stopband performance and computational efficiency compared with the MDISAA-SFW.

## Figures and Tables

**Table 1:** The per iteration computational complexities of different algorithms.

Algorithm	Number of (I)FFT	Complexity
MM-WeCorr-acc	$6M^{2}+8M$	O((12M+16)MNlog2N)
MM-Corr-acc	4M	O(8MNlog2N)
MDISAA-SFW	3M	O(6MNlog2N)
Gra-WeCorr-SFW	$5M^{2}+6M$	O((10M+12)MNlog2N)
Gra-Corr-SFW	4M	O(8MNlog2N)

**Table 2:** Simulation parameters of different design problems.

	λ	Correlation weights ${\left\{w_{k}\right\}}_{k=1-N}^{N-1}$	Stopband $\Omega_{f}$
Case 1${}^{a}$	0	$w_{k}=1,k=1-N,...,N-1$	
Case 2${}^{b}$	0	$w_{k}=\left\{\begin{array}{cc} 1, & k=0\\ 1, & k\in [-80,-51]\cup [51,80]\\ 0, & \mathrm{otherwise} \end{array}\right.$	
Case 3${}^{c}$	0.9	$w_{k}=1,k=1-N,...,N-1$	$\begin{array}{c} (0.04,0.21),(0.23,0.25),(0.28,0.37),\\ \left(0.39,0.49),(0.52,0.56\right) \end{array}$

a: CSS design; b: waveform design with zero correlation zone; c: OWS design with stopband constraint.

**Table 3:** The comparison of the performance parameters between MM-Corr-acc and Gra-Corr-SFW.

	MM-Corr-acc			Gra-Corr-SFW		
(M,N)	t(s)	$N_{I}$	$P_{sum}\left(\mathrm{dB}\right)$	t(s)	$N_{I}$	$P_{sum}\left(\mathrm{dB}\right)$
M=3,N=256	1.249	1687	−63.8	0.071	239	−65.9
M=6,N=512	0.914	496	−68.7	0.110	132	−71.4
M=9,N=768	1.558	460	−72.0	0.178	131	−74.7
M=12,N=1024	2.531	484	−74.4	0.265	132	−77.5
M=15,N=1280	4.483	587	−76.1	0.343	138	−79.1

**Table 4:** The comparison of the performance parameters between MM-WeCorr-acc and Gra-WeCorr-SFW $\left(\varepsilon =10^{-10}\right)$.

	MM-WeCorr-acc			Gra-WeCorr-SFW		
(M,N)	t(s)	$N_{I}$	$P_{zcz}\left(\mathrm{dB}\right)$	t(s)	$N_{I}$	$P_{zcz}\left(\mathrm{dB}\right)$
M=3,N=256	0.584	335	−167.5	0.248	164	−168.2
M=6,N=512	2.691	407	−178.1	1.348	176	−179.5
M=9,N=768	10.167	445	−184.5	5.149	178	−186.3
M=12,N=1024	37.760	486	−189.7	15.106	179	−191.5
M=15,N=1280	84.759	549	−193.5	30.683	182	−195.1

**Table 5:** The comparison of the performance parameters between MDISAA-SFW and Gra-Corr-SFW.

Algorithm	N	$P_{\mathrm{ac}}\left(\mathrm{dB}\right)$	$P_{\mathrm{cc}}\left(\mathrm{dB}\right)$	$P_{\mathrm{sp}}\left(\mathrm{dB}\right)$	ISL	$N_{I}$	t(s)
MDISAA-SFW	N=256	−13.6	−13.9	−17.5	893,573	11932	2.512
	N=512	−13.9	−16.6	−17.9	3,532,447	18,602	6.045
	N=1024	−14.1	−19.3	−18.2	14,013,417	27,268	14.080
Gra−Corr−SFW	N=256	−12.5	−15.9	−20.3	832,482	1216	0.274
	N=512	−12.9	−18.6	−22.2	3,321,729	2100	1.013
	N=1024	−13.3	−21.1	−23.8	13,237,903	3577	3.033

## Abbreviations

The following abbreviations are used in this manuscript:

MIMO	multiple-input multiple-output
(I)FFT	(inverse) fast Fourier transform
CSS	complementary sets of sequences
OWS	orthogonal waveform set
CAN	cyclic algorithm-new
WeCAN	weighted cyclic algorithm-new
LBFGS	limited-memory Broyden, Fletcher, Goldfarb and Shanno
MM	majorization-minimization
MM-Corr	MM-correlation
MM-Corr-acc	MM-correlation-acceleration
MM-WeCorr	MM-weighted correlation
MM-WeCorr-acc	MM-weighted correlation-acceleration
CDMA	code-division multiple-access
OFDM	orthogonal frequency division multiplexing
SFW	sparse frequency waveforms
HFSWR	high frequency surface wave radar
UWB	ultra-wide bandwidth
MDISAA-SFW	multi-dimensional iterative spectral approximation algorithm-SFW
PSD	power spectrum density
Gra-WeCorr-SFW	gradient-weighted correlation-SFW
Gra-Corr-SFW	gradient-correlation-SFW
CISL	complementary integrated sidelobe level

## Appendix A

**Proof.** Due to the unimodular constraint, the energy of each waveform is *N*, i.e., $r_{ii}\left(0\right)=N,\;\;i=1,...,M$. Then, (3) can be rewritten as:

(A1) $$\mathrm{CISL}=\sum_{k=1-N}^{N-1}{\left|\sum_{m=1}^{M}r_{mm}\left(k\right)\right|}^{2}-{\left|\sum_{m=1}^{M}r_{mm}\left(0\right)\right|}^{2}=\sum_{k=1-N}^{N-1}{\left|\sum_{m=1}^{M}r_{mm}\left(k\right)\right|}^{2}-M^{2}N^{2}.$$

Since ${\left|\sum_{m=1}^{M}r_{mm}\left(k\right)\right|}^{2}=\sum_{i=1}^{M}\sum_{j=1}^{M}r_{ii}^{*}\left(k\right)r_{jj}\left(k\right)$, we have:

(A2) $$\mathrm{CISL}=\sum_{i=1}^{M}\sum_{j=1}^{M}\sum_{k=1-N}^{N-1}r_{ii}^{*}\left(k\right)r_{jj}\left(k\right)-M^{2}N^{2}=\sum_{i=1}^{M}\sum_{j=1}^{M}r_{ii}^{H}r_{jj}-M^{2}N^{2}.$$

by substituting (6) into (A2), CISL can be expressed as:

(A3) $$\mathrm{CISL}=\frac{1}{2N}\sum_{i=1}^{M}\sum_{j=1}^{M}p_{ii}^{H}p_{jj}-M^{2}N^{2}.$$

Assume $f_{m}$ is the 2N-point discrete Fourier transform (DFT) vector of the waveform $x_{m},m=1,...,M$, i.e.,

(A4) $$f_{m}\left(i\right)=\sum_{n=1}^{N}x_{m}\left(n\right)e^{-jn\omega_{i}},\omega_{i}=\frac{2\pi }{2N}i,i=1,...,2N,$$

then the PSD vector $p_{ij}$ can be written as:

(A5) $$p_{ij}=f_{i}\circ f_{j}^{*}.$$

According to (A5), it is easy to verify that $p_{ii}^{H}p_{jj}=p_{ij}^{H}p_{ij}$. Thus, (A3) can be denoted as:

(A6) $$\mathrm{CISL}=\frac{1}{2N}\sum_{i=1}^{M}\sum_{j=1}^{M}p_{ij}^{H}p_{ij}-M^{2}N^{2}.$$

Let $w_{t}\left(k\right)=1,k=1-N,...,N-1$, then we have $D_{t}=\mathrm{Diag}\left(1_{2N}\right)=I_{2N}$. Thus, (8) can be simplified as:

(A7) $$\psi_{1}=\frac{1}{2N}\sum_{i=1}^{M}\sum_{j=1}^{M}p_{ij}^{H}p_{ij}-MN^{2}.$$

From (A6) and (A7), we can observe that the only different between CISL and $\psi_{1}$ is the constant term, i.e., CISL and $\psi_{1}$ are equivalent. The proof is complete. ☐

## Appendix B

**Proof.** According to (A5), it is easy to verify that:

(A8) $$p_{im}=p_{mi}^{*}.$$

by substituting (A8) into ∑i=1i≠mMpimHQ′pim, we have:

(A9) ∑i=1i≠mMpimHQ′pim=∑i=1i≠mMpmiTQ′pmi*=∑i=1i≠mMpmiHQ′TpmiT=∑i=1i≠mMpmiHQ′Tpmi.

Since:

(A10) $${Q^{\prime }}^{H}=\frac{1}{4N^{2}}\left(1-\lambda \right){\left(FD_{t}F^{H}\right)}^{H}=Q^{\prime },$$

we have:

(A11) $${Q^{\prime }}^{T}={\left({Q^{\prime }}^{H}\right)}^{*}={Q^{\prime }}^{*}.$$

Thus, (A9) can be rewritten as:

(A12) ∑i=1i≠mMpimHQ′pim=∑i=1i≠mMpmiHQ′*pmi.

The proof is complete. ☐

## Appendix C

**Proof.** Here, we temporarily replace the subscripts i,j with p,q (i.e., $p_{pq}^{l+1}$) to avoid the confusion of the number *j* and the imaginary unit *j*. According to (A4), (A5) and (40), the *k*-th element of $p_{pq}^{l+1}$ can be written as:

(A13) $$p_{pq}^{l+1}\left(k\right)=f_{p}^{l+1}\left(k\right){\left(f_{q}^{l+1}\left(k\right)\right)}^{*}=\sum_{n=1}^{N}\sum_{m=1}^{N}x_{p}^{l}\left(n\right){\left(x_{q}^{l}\left(m\right)\right)}^{*}\cdot e^{j\mu \left(d_{p}^{l}\left(n\right)-d_{q}^{l}\left(m\right)\right)}\cdot e^{-j\frac{2\pi }{2N}\left(n-m\right)k},$$

where $x_{p}^{l}\left(n\right),d_{p}^{l}\left(n\right)$ denote the *n*-th elements of $x_{p}^{l}$ and $d_{p}^{l}$. By using the Taylor series, the expansion of $e^{j\mu \left(d_{p}^{l}\left(n\right)-d_{q}^{l}\left(m\right)\right)}$ in (A13) (which we keep the first three terms) is given by:

(A14) ejμdpl(n)−dql(m)≈1+jμdpl(n)−dql(m)+jμdpl(n)−dql(m)2jμdpl(n)−dql(m)222.

Thus, $p_{pq}^{l+1}\left(k\right)$ can be approximated as:

(A15) $$\begin{array}{cc} p_{pq}^{l+1}\left(k\right)\approx & \sum_{n=1}^{N}\sum_{m=1}^{N}x_{p}^{l}\left(n\right){\left(x_{q}^{l}\left(m\right)\right)}^{*}\cdot e^{-j\frac{2\pi }{2N}\left(n-m\right)k}\\ & +j\mu \sum_{n=1}^{N}\sum_{m=1}^{N}x_{p}^{l}\left(n\right){\left(x_{q}^{l}\left(m\right)\right)}^{*}\cdot \left(d_{p}^{l}\left(n\right)-d_{q}^{l}\left(m\right)\right)\cdot e^{-j\frac{2\pi }{2N}\left(n-m\right)k}\\ & -\frac{\mu^{2}}{2}\sum_{n=1}^{N}\sum_{m=1}^{N}x_{p}^{l}\left(n\right){\left(x_{q}^{l}\left(m\right)\right)}^{*}\cdot {\left(d_{p}^{l}\left(n\right)-d_{q}^{l}\left(m\right)\right)}^{2}\cdot e^{-j\frac{2\pi }{2N}\left(n-m\right)k} \end{array}$$

Let:

(A16) $$f_{i}=F\left(x_{i}^{l}\right),{f^{\prime }}_{i}=F\left(x_{i}^{l}\circ d_{i}^{l}\right),{f^{\prime \prime }}_{i}=F\left(x_{i}^{l}\circ d_{i}^{l}\circ d_{i}^{l}\right).$$

Then, (A15) can be rewritten as:

(A17) $$\begin{array}{cc} p_{pq}^{l+1}\left(k\right)\approx & f_{p}\left(k\right)f_{q}^{*}\left(k\right)+j\mu \left({f^{\prime }}_{p}\left(k\right)f_{q}^{*}\left(k\right)-f_{p}\left(k\right){\left({f^{\prime }}_{q}\left(k\right)\right)}^{*}\right)\\ & -\frac{\mu^{2}}{2}\left({f^{\prime \prime }}_{p}\left(k\right)f_{q}^{*}\left(k\right)+f_{p}\left(k\right){\left({f^{\prime \prime }}_{q}\left(k\right)\right)}^{*}-2{f^{\prime }}_{p}\left(k\right){\left({f^{\prime }}_{q}\left(k\right)\right)}^{*}\right), \end{array}$$

where $f_{p}\left(k\right),f_{p}^{\prime }\left(k\right),f_{p}^{\prime \prime }\left(k\right)$ are the *k*-th elements of $f_{p},{f^{\prime }}_{p},{f^{\prime \prime }}_{p}$. By representing (A17) as a vector, we have:

(A18) $$\begin{array}{cc} p_{pq}^{l+1}\approx & f_{p}\circ f_{q}^{*}+j\left({f^{\prime }}_{p}\circ f_{q}^{*}-f_{p}\circ {f^{\prime }}_{q}^{*}\right)\mu \\ & -\frac{1}{2}\left({f^{\prime \prime }}_{p}\circ f_{q}^{*}+f_{p}\circ {f^{\prime \prime }}_{q}^{*}-2{f^{\prime }}_{p}\circ {f^{\prime }}_{q}^{*}\right)\mu^{2}. \end{array}$$

The proof is complete. ☐
